# The C-terminal selenenylsulfide of extracellular/non-reduced thioredoxin reductase endows this protein with selectivity to small-molecule electrophilic reagents under oxidative conditions

**DOI:** 10.3389/fmolb.2024.1274850

**Published:** 2024-03-08

**Authors:** Huijun Qin, Chenchen Guo, Bozhen Chen, Hui Huang, Yaping Tian, Liangwei Zhong

**Affiliations:** ^1^ Medical School, University of Chinese Academy of Sciences, Beijing, China; ^2^ School of Chemistry and Chemical Engineering, University of Chinese Academy of Sciences, Beijing, China; ^3^ Chinese PLA General Hospital (301 Hospital), Beijing, China

**Keywords:** thioredoxin reductase, selenocysteine, site-directed mutagenesis, protein complex, computer modeling, protein chemistry

## Abstract

Mammalian cytosolic thioredoxin reductase (TrxR1) serves as an antioxidant protein by transferring electrons from NADPH to various substrates. The action of TrxR1 is achieved via reversible changes between NADPH-reduced and non-reduced forms, which involves C-terminal selenolthiol/selenenylsulfide exchanges. TrxR1 may be released into extracellular environment, where TrxR1 is present mainly in the non-reduced form with active-site disulfide and selenenylsulfide bonds. The relationships between extracellular TrxR1 and tumor metastasis or cellular signaling have been discovered, but there are few reports on small-molecule compounds in targeted the non-reduced form of TrxR1. Using eight types of small-molecule thiol-reactive reagents as electrophilic models, we report that the selenenylsulfide bond in the non-reduced form of TrxR1 functions as a selector for the thiol-reactive reagents at pH 7.5. The non-reduced form of TrxR1 is resistant to hydrogen peroxide/oxidized glutathione, but is sensitive to certain electrophilic reagents in different ways. With 5,5′-dithiobis-(2-nitrobenzoic acid) (DTNB) and S-nitrosoglutathione (GSNO), the polarized selenenylsulfide bond breaks, and selenolate anion donates electron to the dynamic covalent bond in DTNB or GSNO, forming TNB-S-Se-TrxR1 complex or ON-Se-TrxR1 complex. The both complexes lose the ability to transfer electrons from NADPH to substrate. For diamide, the non-reduced TrxR1 actually prevents irreversible damage by this oxidant. This is consistent with the regained activity of TrxR1 through removal of diamide *via* dialysis. Diamide shows effective in the presence of human cytosolic thioredoxin (hTrx1), Cys residue(s) of which is/are preferentially affected by diamide to yield disulfide, hTrx1 dimer and the mixed disulfide between TrxR1-Cys497/Sec498 and hTrx1-Cys73. In human serum samples, the non-reduced form of TrxR1 exists as dithiothreitol-reducible polymer/complexes, which might protect the non-reduced TrxR1 from inactivation by certain electrophilic reagents under oxidative conditions, because cleavage of these disulfides can lead to regain the activity of TrxR1. The details of the selective response of the selenenylsulfide bond to electrophilic reagents may provide new information for designing novel small-molecule inhibitors (drugs) in targeted extracellular/non-reduced TrxR1.

## 1 Introduction

Mammalian thioredoxin reductases (TrxRs) (EC 1.6.4.5) are involved in a broad range of physiological functions and are critical for cell growth (Arnér and Holmgren, 2000; Karunanithi et al., 2021; Muri and Kopf, 2023). These reductases are homodimeric selenoproteins. The second residue from the C-terminus is a selenocysteine (Sec498), which forms a selenenylsulfide with a neighboring Cys497 residue under non-reducing conditions (Zhong et al., 1998; Biterova et al., 2005). In molecular architecture, the Cys497/Sec498 couple (numbers assigned based on rat cytosolic TrxR) in one subunit communicates with a redox-active Cys59/Cys64 couple in the opposite subunit, participating in electron transfer from NADPH to substrates (Zhong et al., 2000; Sandalova et al., 2001). Thus, the redox states of the Cys497/Sec498 couple and the Cys59/Cys64 couple are linked to the ratio of NADPH/NADP^+^. Since the levels of cytosolic NADPH are higher than those of NADP^+^ (Pollak et al., 2007), TrxR in the cytosolic compartment is present mainly in NADPH-reduced form. In this article, name of NADPH-reduced TrxR1 is used to distinguish the TrxR reduced by other reducing agents. The functions of NADPH-reduced TrxR have been previously investigated in numerous studies.

Cytosolic TrxR (TrxR1) is also located on the cell membrane and may be released from cells into extracellular environment (Söderberg et al., 2000; Sun et al., 2014; Zhang et al., 2016; Léveillard and Aït-Ali, 2017). The oxidizing nature of the extracellular environment is vastly different from the highly reducing nature of the intracellular compartment (Ottaviano et al., 2008). By breaking cells/tissues, TrxR1 was purified from the released cytosolic proteins (Arnér et al., 1999). The purified extracellular TrxR1 has been confirmed to contain active-site disulfide and selenenylsulfide bonds (Zhong et al., 1998; Zhong et al., 2000; Lee et al., 2000), here called as non-reduced form of TrxR1 to avoid conceptual confusion with the oxidized form of TrxR1, which was induced by oxidative stress (Xu et al., 2015).

The extracellular TrxR1 and extracellular thioredoxin (Trx) were found to alter the balance between the tissue inhibitors of metalloproteinases (TIMP) and matrix metalloproteinases (MMP) (Farina et al., 2001). This balance was potentially relevant to tumor invasion and metastasis (Hofmann et al., 2000). In addition, extracellular TrxR1/Trx were related to cell signaling (Léveillard and Aït-Ali, 2017), inflammation and tumor progression (Söderberg et al., 2000). New functions of the non-reduced TrxR1 in extracellular or oxidizing environments are gradually being revealed. Interestingly, the activity of serum TrxR was disrupted by electrophilic reagents, such as auranofin (Zhang et al., 2016). The latter has been known to bind with high affinity to thiol and selenol groups. Therefore, we are considering which thiol/selenol-reactive reagents can react with the non-reduced form of TrxR1? The direct question is: how do thiol/selenol-reactive molecules react with selenenylsulfide bond? During the process of revising this article, the latest report showed it was possible about selenenylsulfide (S-Se) bond oxidative addition to auranofin derivatives (Dos Santos and Paschoal, 2024). However, the mechanisms underly inhibition of serum/non-reduced TrxR by different electrophiles remain largely unclear.

Elevated level of reactive oxygen species (ROS) has been detected in cancer cells (Moloney and Cotter, 2018; Nakamura and Takada, 2021), inevitably causing a redox shift of reduced TrxR to the non-reduced form. To maintain redox balance, the expression of TrxR1 was high in some cancer cells (Lincoln et al., 2003; Dong et al., 2016; Wu et al., 2021). The activity of plasma TrxR in the patients with gastric cancer, non-small cell lung cancer and hepatocellular carcinoma was significantly higher than in healthy controls (Peng et al., 2019; Ye et al., 2019; Zenlander et al., 2023). Thus, the non-reduced form of TrxR1 might be a part of cancer cell growth, invasion and migration. Although NADPH-reduced TrxR1 and its substrate cytosolic thioredoxin (Trx1) have been investigated as potential target(s) for anti-cancer therapies (Zhang et al., 2019; Busker et al., 2020; Zhang et al., 2021; Gencheva and Arnér, 2022; Patwardhan et al., 2022), few compounds have been found to exclusively inhibit the non-reduced form of TrxR1 while leaving the reduced form of TrxR1 less affected. The unique properties afforded by the non-reduced form of TrxR1 remain unclear.

Currently, redox modulators are considered to be a novel class of promising anti-cancer drugs (Wondrak, 2009; Hu et al., 2020). It would therefore be particularly interesting to understand the responses of the non-reduced TrxR1 to redox modulators or small-molecule electrophilic inhibitors. Theoretically, an electrophile reacts with a nucleophile by accepting electrons. The thiolate/selenolate anion is one of the strongest biological nucleophiles. In this study, we utilized eight types of small-molecule thiol-reactive reagents as electrophilic models, and primarily focused on the non-reduced form of TrxR1. We report that the non-reduced TrxR1 varies in its response to different electrophilic reagents. This arises from whether the selenenylsulfide (S-Se bond) in the non-reduced TrxR1 easily reacts with electrophiles. The S-Se bond is polarized at pH 7.5, the electron density moves away from the S atom towards the Se atom, resulting in a nucleophilic Se atom. With electrophilic reagents, the polarized S-Se bond breaks, and donates electron pair to electrophile, forming a new bond. Our results can provide valuable information for designing novel small-molecule drugs in targeted extracellular/non-reduced TrxR1.

## 2 Experimental procedures

### 2.1 Reagents and materials

Reduced/oxidized glutathione (GSH/GSSG), H_2_O_2_, diamide, 5,5′-dithiobis-(2-nitrobenzoic acid) or DTNB, iodoacetamide (IAM), iodoacetic acid (IAA), AldrithiolTM-4 and 1-methylpropyl 2-imidazolyl disulfide (Px-12) were purchased from Sigma-Aldrich Co. (St Louis, MO, United States). S-nitrosoglutathione (GSNO) was kindly provided by Professor Chang Chen, Institute of Biophysics, Chinese Academy of Sciences. The monoclonal antibody against TrxR1 was purchased from Santa Cruz Biotechnology (Germany). Horseradish peroxidase (HRP)-conjugated secondary antibodies were purchased from Invitrogen (United States). Mammalian TrxR1 was purified from calf liver as described previously (Zhong et al., 1998; Arnér et al., 1999). Human cytosolic Trx (hTrx1) was purified according to a previously described method (Zhai and Zhong, 2010). Human serum samples were the remaining portion of the previous study (Bai et al., 2020). Ethics Committee of Chinese PLA General Hospital approved this procedure. Informed consent was obtained from all participants. The study was performed in conformance with the Declaration of Helsinki ethical guidelines.

### 2.2 Construction, expression, and purification of site-directed mutants of TrxR1

The replacement of Sec498 by Cys and deletion of C-terminal Sec498 and Gly499 were performed according to a previously described method (Zhong and Holmgren, 2000). The plasmid expressing the Sec498Cys mutant of TrxR1 was used to generate other mutants with different mutations. The QuickChange Site-Directed Mutagenesis Kit (Agilent Technologies, Inc., CA, United States) was used to develop point mutations. To replace TrxR1-Cys189 with Ser, the forward primer was 5′-T TAC TCC CCG GGG AAG ACC CTA GTG GTT GG-3′, and the reverse primer was 5′- C CGG GGA GTA AGG CAA GGA GAA AAG ATC GT-3’. To replace TrxR1-Cys296 with Ser, the forward primer was 5′- G TTG CTT GCA GTA GGA AGA GAT TCT ACA AGA A-3′, and the reverse primer was 5′- T CTC TAA GCC AAT AGT TCT TGT AGA ATC TCT T-3’. To replace TrxR1-Cys458 with Ser, the forward primer was 5′- A CTC AAG AGC GGG CTG ACC AAG CAG CAG-3′, and the reverse primer was 5′- AGC CCG CTC TTG AGT GCG GCT GCA AAG CC-3’. To replace TrxR1 Cys497 and Sec498 with Ser, the forward primer was 5′-GAC ATC CTC CAG TCT GGC TCC TCC GGT TAA GCC-3′, and the reverse primer was 5′-CCA CAC TGG GGC TTA ACC GGA GGA GCC AGA-3’. We constructed point mutations for four sites (Cys189/296/458Ser/Sec498Cys) though several rounds of PCR with the corresponding primer sets mentioned above. The underlined bases in the primers are the mutated sites. Each mutant construct was sequenced to confirm the correct mutation(s). *Escherichia coli* strain Rosetta (DE3) was used to express the recombinant proteins. The bacterial cultures were grown under shaking at 37°C until the cell density reached an OD600 of ∼1.0. At this time, IPTG was added at a final concentration of 0.5 mM, and protein expression was induced for 4 h at 37°C. Cells were harvested by centrifugation and used to purify different recombinant TrxR1 variants as previously described (Zhong and Holmgren, 2000). All mutants of TrxR1 were purified to homogeneity. These include the Sec498Cys mutant, DesSec498Gly499 truncated TrxR1, Cys189Ser/Sec498Cys mutant, Cys296Ser/Sec498Cys mutant, Cys458Ser/Sec498Cys mutant, Cys497Ser/Sec498Ser mutant, and tetra Cys189/296/458Ser/Sec498Cys mutant TrxR1.

### 2.3 Activity analysis of the non-reduced TrxR1 after treatments with thiol-reactive reagents

The activity of TrxR1 was measured using an insulin reduction assay (Arnér et al., 1999) in a Double Beam UV/VIS spectrophotometer (UV-8500, Techcomp, Shanghai, China). In this assay system, TrxR1 transfers electrons from NADPH to hTrx1 to yield a reduced form of hTrx1, which in turn reduces insulin. Thus, TrxR1 activity was monitored by detecting the decrease in absorbance at 340 nm because of the consumption of NADPH. The activity was expressed as change in absorbance at 340 nm per min per mg of protein. To test the effects of thiol-reactive reagents on the activity of TrxR1, the non-reduced TrxR1 (30 μL of 7.5 μM) was pre-incubated with 10 mM H_2_O_2_, diamide, DTNB, GSNO, GSSG, IAM, Aldrithiol™-4 or 2 mM Px-12 at room temperature for 30 min. The pre-treated TrxR1 (7.5 μL) was added to 500 μL of the assay mixture to initiate the reaction. The assay mixture contained 50 mM potassium phosphate buffer, pH 7.5, 1 mM EDTA (PE buffer), 0.2 mM NADPH, 5 μM hTrx1 and 160 μM insulin. Before and after removal of the small-molecule reagents *via* dialysis, the remaining activity of TrxR1 was measured respectively using the same method. Since the structure of DTNB is similar to Aldrithiol™-4, and both of them exhibited inhibitory effects on the non-reduced TrxR1, a more commonly used DTNB was chosen in subsequent studies.

### 2.4 Identification of DTNB-reactive group(s) in the non-reduced TrxR1 by thiol quantification assay and site-directed mutagenesis of surface Cys residues

The method described by Ellman (Ellman, 1959; Shetab-Boushehri, 2018) was used. A cuvette containing the non-reduced TrxR1 (1.31 μM) in PE buffer was mixed with 1.5 mM DTNB in a total volume of 205 μL. At room temperature, the reaction was followed for 5 min by monitoring the increase in absorbance at 412 nm due to the formation of 5-thio-2-nitrobenzoic acid (TNB^-^) using a Double Beam UV/VIS Spectrophotometer (UV-8500). TNB^−^ is an excellent leaving group with a thiol p*K*_a_ of 4.5. In theory, the reaction of each thiol/selenol with DTNB gives one TNB^−^. The content of the generated TNB^−^ was calculated from the molar extinction coefficient of TNB^−^ (ε412 = 13,700 M^-1^cm^-1^). The experiments were repeated at least three times.

To determine which residue mutation will affect the production of TNB^−^, the following experiments were performed in 96-well microplates using a microplate reader (Multiskan MK3, Thermo, United States). In total, an aliquot (10 μL, or 4 μg) of the non-reduced TrxR1, Cys497Ser/Cys498Ser mutant of TrxR1, Cys458Ser mutant of TrxR1 or Cys189/296/458Ser mutant of TrxR1 was added to the wells. The protein samples in each well were then mixed with 200 μL of 2 mM DTNB. After incubation for 30 min at room temperature, the change in absorbance at 412 nm was recorded. The control wells contained PE buffer instead of protein samples. The analysis was repeated three times for each protein.

### 2.5 Computational analysis

All calculations were performed using Gaussian 09 Programs. The geometries in the gas-phase were fully optimized at the Density Functional Theory (DFT) B3LYP/6-31+G (d, p) level (Vosko et al., 1980; Lee et al., 1988; Becke, 1993; Ashvar et al., 1996). The adiabatic ionization potential (AIP), the adiabatic electron affinity (EAad) and all energies (not including the zero-point energies) in H2O solvent given in this paper were calculated by performing B3LYP/6-311++G (d, p) single-point energy calculations at the optimized geometries from the B3LYP/6-31+G (d, p) calculations in gas-phase using the polarized continuum model (PCM) (Miertus et al., 1981; Cossi et al., 2002; Cossi et al., 2003) in the Self-Consistent Reaction Field (SCRF) theory.

### 2.6 Detection of diamide-induced TrxR1-hTrx1 complex by non-reducing SDS-PAGE

As purified hTrx1 may form homodimer via disulfide bond between Cys73 residues of two monomers during storage, the stored hTrx1 was incubated with DTT to reduce disulfide bonds, followed by removal of DTT through dialysis. The freshly treated hTrx1 has free thiol on surface Cys73 (Qin et al., 2015). Then, the freshly treated hTrx1 (twice the concentration of TrxR1) was incubated with the non-reduced TrxR1 at room temperature in the presence of 10 mM diamide. After 30 min of incubation, the reaction was stopped by adding equal volumes of 2 × SDS sample buffer without any reducing reagents. After being heated for 10 min at 95°C, the resulting sample was subjected to non-reducing SDS-PAGE and protein bands in the gel were stained with Coomassie Brilliant Blue.

### 2.7 Effect of thiol-reactive reagents on diamide-induced formation of TrxR1-hTrx1 complex

The non-reduced TrxR1 was, respectively, pre-incubated with 10 mM H_2_O_2_, diamide, GSSG, GSNO, DTNB, IAM or IAA for 30 min at room temperature. After removal of the excess reagents via dialysis, the pre-treated TrxR1 was mixed with hTrx1 in the presence of 10 mM diamide. The other procedures were identical to those described in Section 2.6.

### 2.8 Effect of site-directed mutagenesis on diamide-induced formation of TrxR1-hTrx1 complex

To identify the site(s) involved in the formation of the TrxR1-hTrx1 complex, the available mutants of TrxR1 were incubated with a 2-fold concentration of hTrx1 at room temperature in the presence of 10 mM diamide. Alternatively, the non-reduced TrxR1 was mixed with the Cys73Ala mutant of hTrx1 in the presence of diamide, because Cys73 is located on the surface of hTrx1 (Hwang et al., 2015). The other procedures were identical to those described in Section 2.6.

### 2.9 Analysis of TrxR1 states in human sera by Western blot

Each serum sample was diluted to give a protein concentration of 4 mg/mL. The diluted samples were separately incubated with or without 10 mM DTT for 30 min, and an aliquot (40 μL) of each sample was then mixed with 10 μL of 5 × SDS sample buffer. After being heated for 10 min at 95°C, the resulting samples were subjected to non-reducing SDS-PAGE. The resolved proteins were then electroblotted to polyvinyl difluoride membranes (Novex, United States). The membranes were subsequently placed in blocking solution containing 1 × 50 Tris-buffered saline (TBS) with 5% w/v skim milk and 0.1% Tween-20 (TBS-T buffer) for 4 h with gentle shaking. The membranes were then incubated with monoclonal antibody raised in mouse against human TrxR1 at a dilution of 1:1,000 overnight at 4°C. The bound primary antibody was detected by HRP-conjugated goat anti-mouse IgG (second antibody at 1:10,000 Dilution), followed by ECL (enhanced chemiluminescence, Millipore, United States) detection according to the manufacturer’s instructions.

## 3 Results

### 3.1 Selective reactions of the non-reduced TrxR1 to thiol-reactive reagents

**FIGURE 1:**
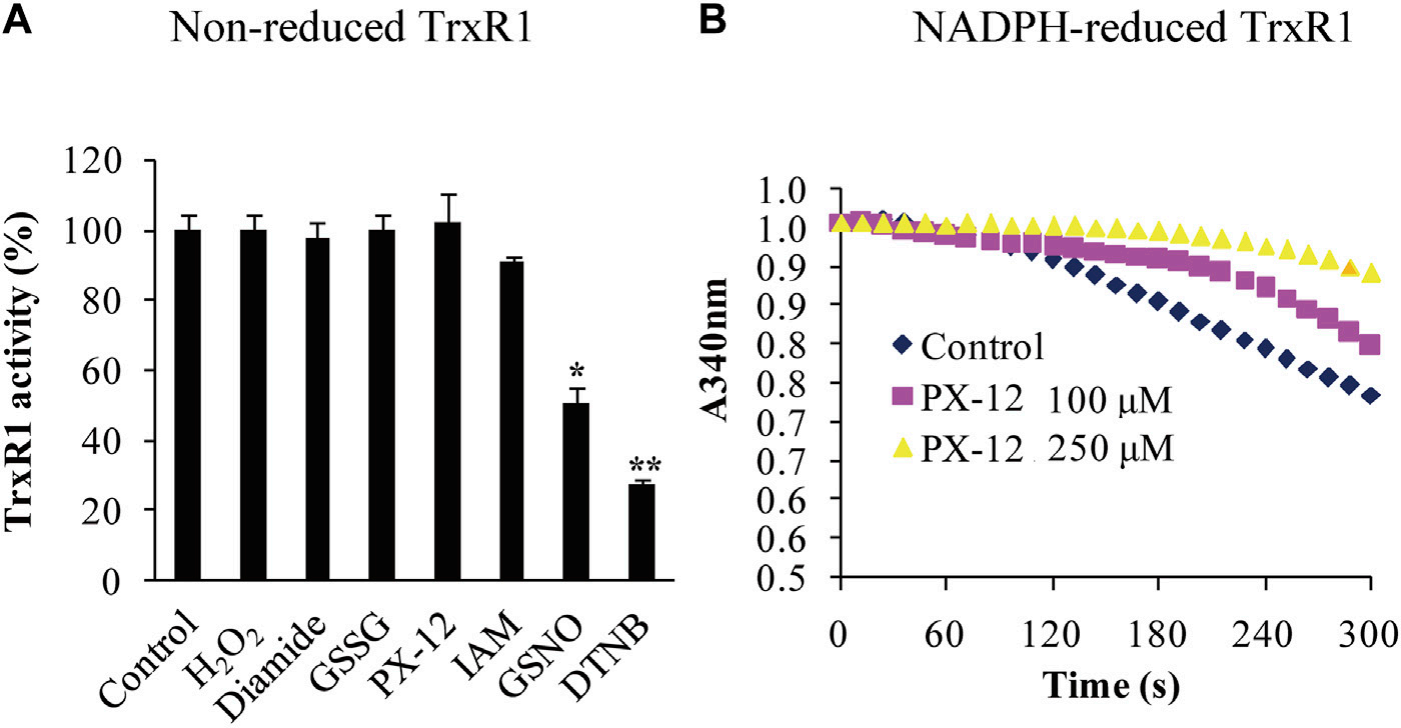
Differential responses of the non-reduced TrxR1 to thiol-reactive reagents. **(A)** Activity analysis of non-reduced TrxR1. The non-reduced form of TrxR1 was pretreated with the indicated thiol-reactive reagents, followed by dialysis to remove these small molecules. The resulting TrxR1 was used to measure its remaining activity. To compare the treated samples with control sample, unpaired Student's t-test was used. Data were presented as the mean ± standard deviation (SD; *n* = 3). *, *p* < 0.05; **, *p* < 0.01. **(B)** Inhibitory effect of Px-12 on NADPH-reduced TrxR1. The inhibition of NADPH-reduced TrxR1 by Px-12 appeared in a dose-dependent manner.

The structures of the thiol-reactive reagents used in this study are shown in Supplementary Table S1. After non-reduced TrxR1 was incubated with 10 mM of H_2_O_2_, diamide, GSSG, IAM or 2 mM of Px-12 at room temperature, pH 7.5 for 30 min, respectively, each sample of the treated TrxR1 was divided into two parts. One part was directly subjected to assay TrxR1 activity, the results were showed in Table 1. The other part was first dialyzed to remove free small-molecule reagents, followed by measuring TrxR activity, the results from which were presented in Table 1; Figure 1A. Among the ineffective reagents, only 10 mM diamide caused a 86.4% decrease in an ability for TrxR1 to transfer electrons from NADPH to hTrx1 (Table 1), this impact was no longer observed after removal of diamide *via* dialysis (Table 1; Figure 1A); IAM, IAA and Px-12 exhibited inhibitory effects on NADPH-reduced TrxR1 (Figure 1B) (Zhong et al., 1998). By contrast, the non-reduced form of TrxR1 was sensitive to GSNO and DTNB. (Since the structure of DTNB is similar to Aldrithiol™-4, and both of them exhibit inhibitory effects on the non-reduced TrxR1, a more commonly used DTNB was chosen in subsequent studies.) The activity of the non-reduced TrxR1 was reduced by 50% or 73%, respectively, after it was incubated with 10 mM GSNO or DTNB for 30 min, followed by removal of excess GSNO or DTNB *via* dialysis (Table 1; Figure 1A). These results indicate that covalent modifications may occur on the non-reduced form of TrxR1 during incubation with GSNO or DTNB. Based on the responses of the non-reduced TrxR1, these thiol-reactive reagents are classified into three types: 1) H_2_O_2_ and GSSG have no inhibitory effect on the non-reduced form of TrxR1, and their reduction can be catalyzed by a NADPH-TrxR1 system (Zhong and Holmgren, 2002) or a NADPH-TrxR1-Trx1 system (Tan et al., 2010); 2) IAM and Px-12 exclusively inhibit the NADPH-reduced form of TrxR1; and 3) DTNB and GSNO are inhibitors of the non-reduced TrxR1 (Figure 1A; Table 1), even if both are substrates of NADPH-TrxR1 system (Holmgren, 1977; Nikitovic and Holmgren, 1996). The distinctive effects of these small-molecule reagents on the non-reduced form of TrxR1 (Figure 1A; Table 1) have generated considerable interest. For instance, the reaction with a thiol/selenol group is a common feature of IAM and DTNB, but an apparent difference between IAM and DTNB was observed in their effect on the non-reduced form of TrxR1 (Table 1; Figure 1A). Furthermore, modification of thiol is a chemical feature of Px-12 (Kirkpatrick et al., 1998) and GSNO (Singh et al., 1996), but they also showed distinctive behaviors in terms of their effects on the non-reduced TrxR1. We sought to determine why the non-reduced form of TrxR1 is resistant to IAM and Px-12, but is sensitive to DTNB or GSNO. We thus attempted to identify the residue(s) that participate(s) in the reaction with DTNB/GSNO.

**TABLE 1:** Effect of thiol-reactive reagents on TrxR1 activity^a^

Thiol-reactive molecule	Relative value of TrxR1 activity (%)	
	Before dialysis	After dialysis
Not added (Control)	100	100 ± 3.3
GSSG	100.4 ± 6.6	100.1 ± 4.3
H_2_O_2_	95.2 ± 1.6	99.5 ± 4.3
IAM	100.4 ± 2.6	91.8 ± 1.1
Px-12	N/T^b^	101.9 ± 8.2
GSNO	37.5 ± 0.03	49.8 ± 4.6
Diamide	13.6 ± 0.1	97.6 ± 4.7
Aldrithiol^TM^-4	5.4 ± 1.6	N/T^c^
DTNB	3.6 ± 1.7	26.5 ± 1.7

^a^ Non-reduced TrxR1 was pre-treated with above molecules, respectively. The activity was determined before or after removing these molecules *via* dialysis.

^b^ Not Tested. It is known that Px-12, modifies hTrx1 to inhibit its activity. So, it is meaningless to measure the reduction of hTrx1 by TrxR1 in the presence of Px-12 (Oblong et al., 1994; Kirkpatrick et al., 1998; Lundberg et al., 2019).

^c^ Since the structure of DTNB is similar to Aldrithiol™-4, and both of them exhibit inhibitory effects on the non-reduced TrxR1, a more commonly used DTNB was chosen in subsequent studies.

### 3.2 Selenenylsulfide of the non-reduced TrxR1 reacted with DTNB

Because three-dimensional structures of the non-reduced TrxR1 are available (Sandalova et al., 2001; Cheng et al., 2009; Fritz-Wolf et al., 2011), we surveyed the surface accessibility of Cys/Sec residues in the non-reduced form of TrxR1. As shown in Supplementary Figure S1, among 14 Cys residues and 1 Sec residue per subunit, Cys189, Cys296, Cys497 and Sec498 are closer to the surface than the others; Cys458 is located at the subunit interface.

**FIGURE 2:**
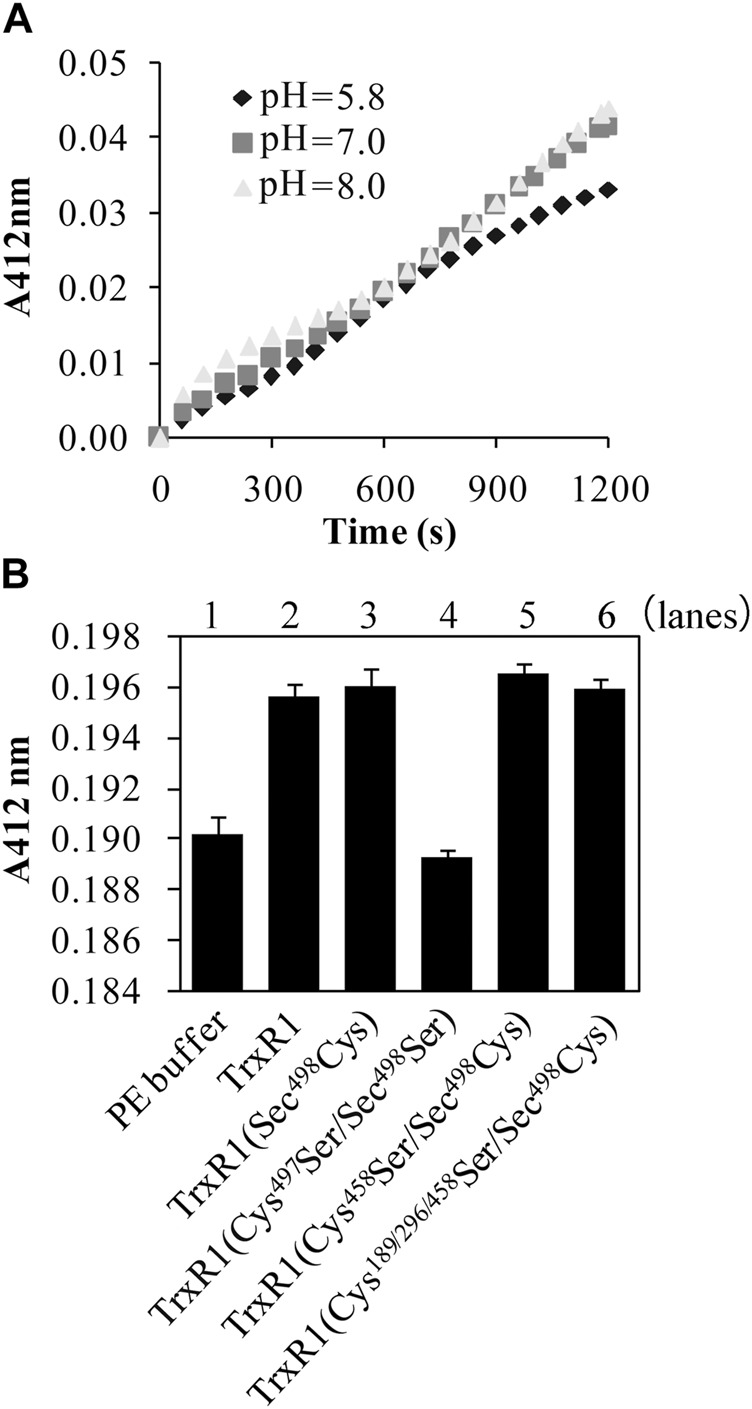
Reaction of the non-reduced TrxR1 or TrxR1 mutants with DTNB in the absence of NADPH. **(A)** Effect of pH on TNB^−^ generation from the reaction of non-reduced TrxR1 with DTNB. **(B)** Contribution of Cys497 and Sec498 to TNB^−^ generation. Only the content of TNB^−^, generated from the reaction of Cys497Ser/Sec498Ser mutant TrxR1 with DTNB, was similar to that from the reaction of PE buffer with DTNB. Each experiment was repeated at least three times.

Using the titration method with DTNB, we detected 1.6 ± 0.2 reactive species per subunit of the non-reduced TrxR1 at pH 7.5. Titration reactions performed at pH 7 or pH 8 generated similar amounts of TNB^−^, which were higher than those produced at pH 5.8 (Figure 2A). To identify the DTNB-reactive species in the non-reduced form of TrxR1, we constructed a series of TrxR1 mutants, including single Sec498Cys and Sec498Ser mutants of TrxR1; a DesSec498Gly499 TrxR1 lacking C-terminal Sec498 and Gly499 residues; double Cys189Ser/Sec498Cys, Cys296Ser/Sec498Cys, Cys458Ser/Sec498Cys and Cys497Ser/Sec498Ser mutants of TrxR1; and a tetra Cys189/296/458Ser/Sec498Cys mutant of TrxR1. Among them, only the double Cys497Ser/Sec498Ser mutant of TrxR1 failed to cause the generation of TNB^−^ when it was incubated with DTNB in the absence of NADPH. The amount of TNB^−^ generated by the reaction of other mutants with DTNB showed similar to that observed with non-reduced TrxR1 in the absence of NADPH. Representative results are shown in Figure 2B. Our result is consistent with 0.1–0.2 DTNB-reactive species per subunit of TrxR1 mutants missing 8 or 16 C-terminal amino acid residues (Fritz-Wolf et al., 2007). These data indicate that C-terminal Cys497 and Sec498 residues allow the non-reduced form of TrxR1 to sense DTNB. Because Cys497 and Sec498 form selenenylsulfide under non-reducing conditions (Zhong et al., 2000; Cheng et al., 2009), this new finding raises the following question: how does selenenylsulfide react with DTNB?

### 3.3 Computational modeling

**FIGURE 3:**
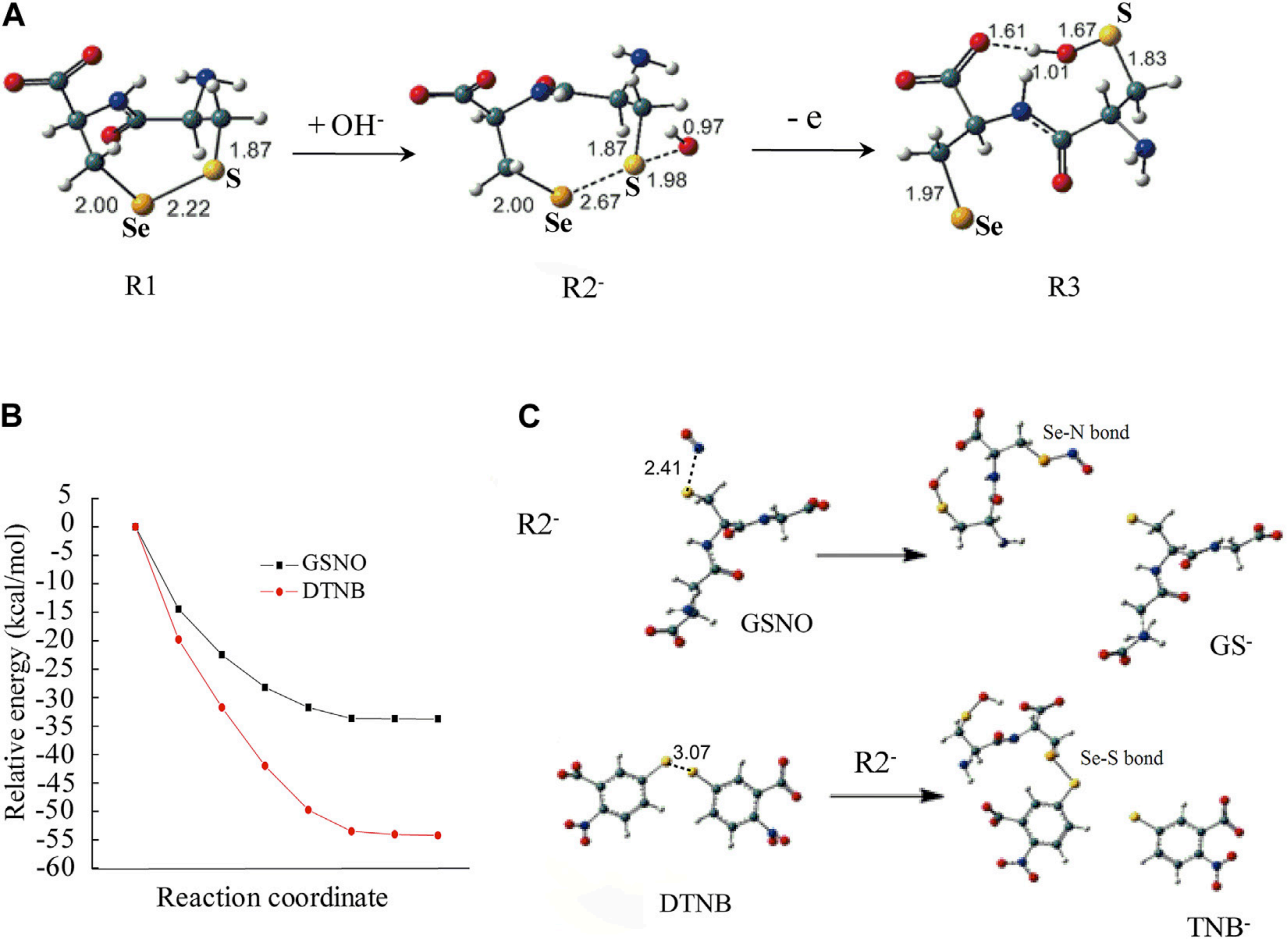
Computational modeling in unraveling the mechanism for selenenylsulfide to react differentially with DTNB, GSNO or GSSG. **(A)** Optimized structures of select important stationary points along with the key distance in angstroms. **(B)** The potential energy curves. All energy values are given in *k*_cal_/mol at the B3LYP/6-311++G(d,p)//B3LYP/6-31+G(d,p) level in the H_2_O solvent. **(C)** The geometry structures of the reactants and products. The geometric structure changes for the reaction between R2^−^ and DTNB or the reaction between R2^−^ and GSNO. Trivial hydrogen atoms are omitted for clarity. Color code: C, green; O, red; Se, orange; S, yellow; N, blue; H, white.

To investigate the possible mechanisms for selenenylsulfide to selective response to DTNB, GSNO or GSSG, a theoretical calculation was carried out using Density Functional Theory. Because the structure of non-reduced TrxR1 is too large to calculate using the Quantum chemistry method, a “H_2_N-Cys497-Sec498-COO-” model containing selenenylsulfide (assigned as R1 in Figure 3A) was used in this calculation.

The results showed that the energy to form the radical cation from R1 was high. However, R1 and OH^−^ could form a complex (R2^−^), which was reversible because the reaction enthalpy was only 1.7 *k*_cal_/mol. As shown in Figure 3A, the selenenylsulfide (S-Se bond) in R2^−^ was longer than that in R1 when OH^−^ approached the S atom, by which the S-Se bond was weaker. The distance between the S atom and the O atom of OH^−^ was 1.98 Å, suggesting weak S···O bonding. Meanwhile, the geometry of R2^−^ was ready to change into R3 with a trans-conformation after losing an electron. In R3, the distance between the H atom of the OH^−^ and the O atom of the carboxyl was 1.61 Å, with the hydrogen bond R3 was stabilized. This open conformation of R3 is similar to the conformation of the C-terminus in the crystal structure of Sec498Cys mutant TrxR1 (Sandalova et al., 2001).

To investigate the possibility of electron transfer from R2^−^ to DTNB, GSNO or GSSG, we calculated the AIP of R2^−^ and the EAad of DTNB, GSNO or GSSG, respectively. The AIP and EAad were evaluated according to the following definition: AIP = E (optimized cation)-E (optimized neutral); EAad = E (optimized neutral)-E (optimized anion). As shown in Table 2, the AIP of R2^−^ was slightly lower than the EAad of DTNB or GSNO, indicating that electron transfer from R2^−^ to DTNB or GSNO is possible. However, the AIP of R2^−^ was higher than the EAad of GSSG, indicating that electron transfer from R2^−^ to GSSG is impossible, which is consistent with the no effect of GSSG on the activity of the non-reduced TrxR1 (Figure 1A).

**TABLE 2:** Adiabatic ionization potential of R2 and adiabatic electron. affinities (EA_ads_) of DTNB, GSNO and GSSG.

R2^-^	DTNB	GSNO	GSSG
AIP	EA_ad_	EA_ad_	EA_ad_
85.1	85.8	85.9	49.8

All energy values are given in *k*_cal_/mol at the B3LYP/6-311++G(d,p)//B3LYP/6-31+G(d,p) level in H_2_O solvent.

We then combined R2^−^ and DTNB as the initial reaction configuration to calculate the potential energy curve and the geometry structures of the reactants and products. The energy continuously decreased along the reaction pathway, indicating that these reactions are energetically favorable (Figure 3B). The structures of the products showed that the S-Se bond was formed between R2^−^ and TNB, the charge distribution showed that the TNB^−^ was generated (the lower panel of Figure 3C), which is in good agreement with the experimental results (Figure 2A). The potential energy curve and the geometry structures of the products for the reaction between R2^−^ and GSNO showed similar response. This reaction process was energetically favorable as well (Figure 3B), and the Se-N bond was formed between R2^−^ and NO, the charge distribution showed that GS^−^ was generated (the upper panel of Figure 3C).

### 3.4 Role of selenenylsulfide in the formation of the TrxR1-hTrx1 complex under oxidative conditions

**FIGURE 4:**
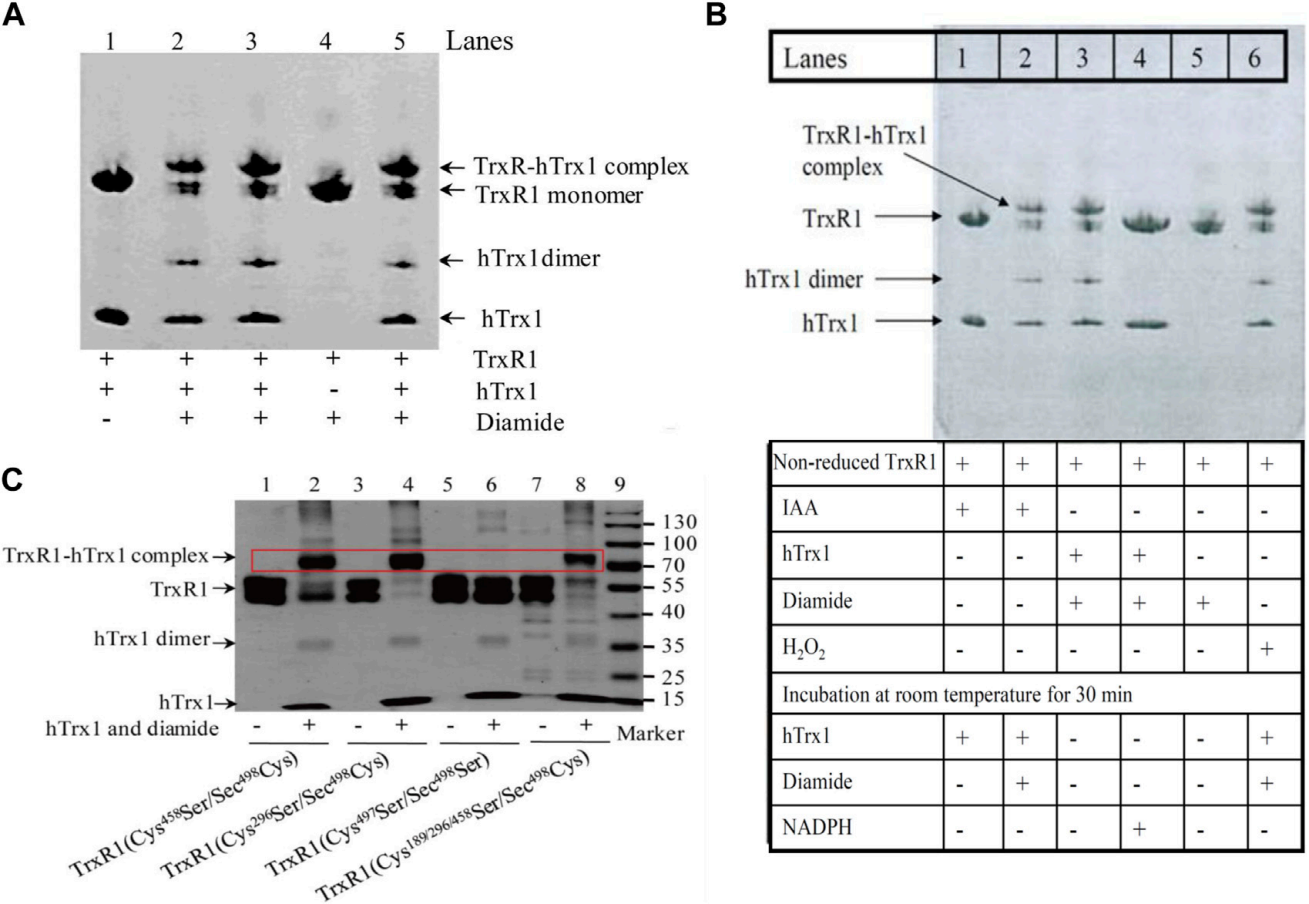
Detection of the complexes between the non-reduced TrxR1 and hTrx1-Cys73 by non-reducing SDS-PAGE. **(A)** Diamide-induced formation of the TrxR1-hTrx1 complex. Lane 1, non-reduced TrxR1 and hTrx1 without diamide as control; lanes 2, 3 and 5, non-reduced TrxR1 and hTrx1 plus diamide; lane 4, diamide treated TrxR1. **(B)** Effect of IAA, H_2_O_2_, or NADPH on diamide-induced formation of the TrxR1-hTrx1 complex. Lane 1, non-reduced TrxR1 pretreated with IAA, then hTrx1 was added; lane 2, the other components were the same as lane 1, except for the addition of diamide; lane 3, the reaction of the non-reduced TrxR1 with hTrx1 in the presence of diamide; lane 4, the other components were the same as lane 3, except for the addition of NADPH; lane 5, incubation of the non-reduced TrxR1 with diamide; lane 6, H_2_O_2_-pretreated TrxR1 was incubated with hTrx1 and diamide. **(C)** Location of the residue(s) in the non-reduced TrxR1 for the connection to hTrx1-Cys73 under oxidizing condition. Non-reducing SDS-PAGE was used to separate the protein mixture. The positions of the TrxR1-hTrx1 complex are marked with the red rectangle box.

In terms of the reaction with DTNB, the selenenylsulfide showed similar behavior to thiol. We then tested whether the non-reduced form of TrxR1 could bind to a free thiol-containing molecule and form a stable complex under oxidative conditions. Therefore, we used the freshly treated hTrx1 (please refer to Section 2.6 for the method), which is known to contain free thiol on its surface (Hwang et al., 2015), as model. The efficiency of TrxR1-hTrx1 complex formation was evaluated in the presence of diamide, H_2_O_2_ (as a source of thiol oxidant) or PE buffer (as control). PE buffer showed no effect (lane 1 in Figure 4A). Diamide caused the formation of the TrxR1-hTrx1 complex as well as hTrx1 dimer (lanes 2,3,5 in Figure 4A, and lane 3 in Figure 4B), but not TrxR1 dimer (lane 4 in Figure 4A, lane 5 in Figure 4B).

**FIGURE 5:**
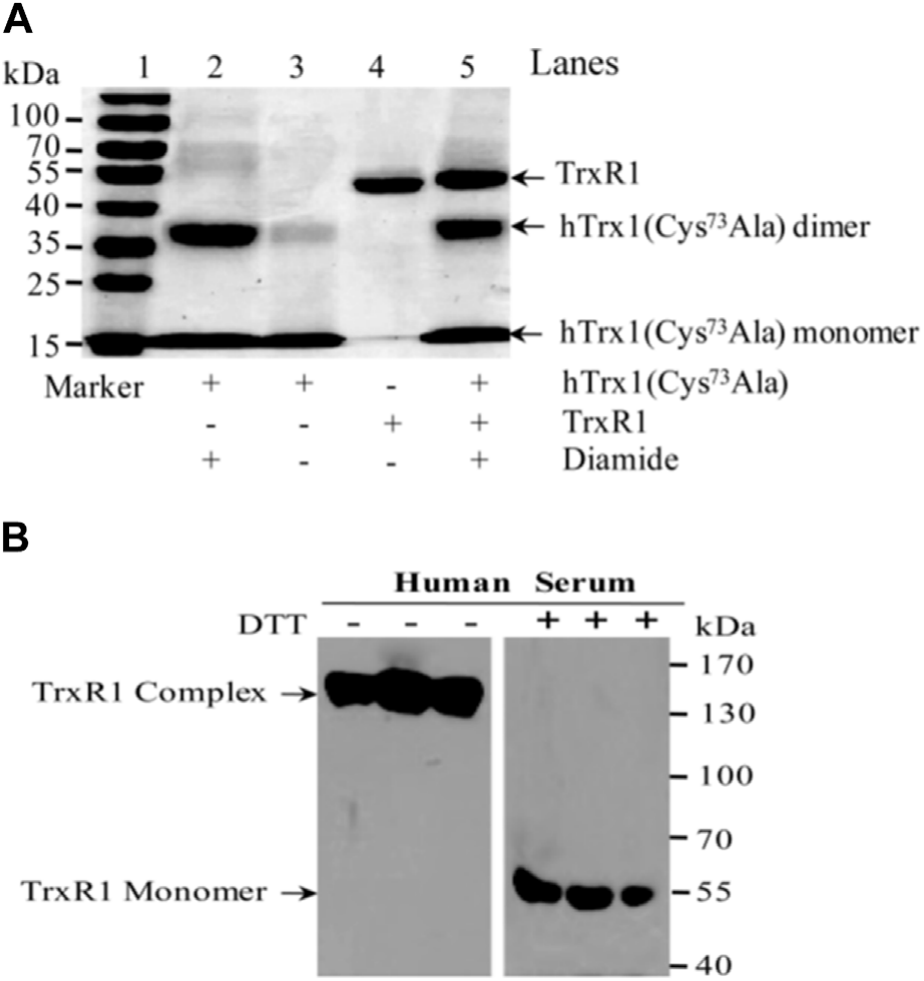
Non-reducing SDS-PAGE and Western blot. **(A)** Non-reducing SDS-PAGE. hTrx1-Cys73 implicated in the connection to the non-reduced TrxR1 under oxidizing condition. Replacement of Cys73 by Ala eliminated the ability of hTrx1 to form the TrxR1-hTrx1 complex (lane 5), but did not affect the formation of the hTrx1 dimer (lane 2) in the presence of diamide. Lane 3, no covalent linkage between two subunits of non-reduced TrxR1 was produced in the presence of diamide. **(B)** Western blot. Redox status of TrxR1 in human sera. Left panel, human serum without DTT treatment. Right panel, human serum with DTT treatment. All of samples were separated by non-reducing SDS-PAGE, followed by Western blotting.

H_2_O_2_ induced the formation of hTrx1 dimer, but not the TrxR1-hTrx1 complex or TrxR1 dimer (Zhong’s observation). Pretreatment of the non-reduced TrxR1 with H_2_O_2_ did not block the formation of the diamide-induced TrxR1-hTrx1 complex (lane 6 in Figure 4B). Similarly, the pretreatment with IAA was unable to block the formation of the diamide-induced TrxR1-hTrx1 complex (lane 2 in Figure 4B); in this regard, the impact of GSSG on the non-reduced TrxR1 is similar to that of IAA and H_2_O_2_ (Table 3). These results confirm that in the non-reduced TrxR1, it is not the selenol/thiol group that reacts with DTNB/GSNO. Moreover, diamide could no longer induce the formation of TrxR1-hTrx1 complex once the non-reduced TrxR1 was pre-incubated with DTNB or AldrithiolTM-4 (Table 3). Further supporting the notion that inactivation of the non-reduced TrxR1 through incubation with DTNB or AldrithiolTM-4 (Table 1) was caused by the reaction of selenenylsulfide with them to form Cys497/Sec498 modification. These findings also suggest that selenenylsulfide is the possible site involved in the formation of the TrxR1-hTrx1 complex. NADPH could break the diamide-induced TrxR1-hTrx1 complex and release TrxR1 and hTrx1 (lane 4 compared with lane 3 in Figure 4B). With DTT, similar results were observed (data not shown). To further confirm that the selenenylsulfide is required to form the diamide-induced TrxR1-hTrx1 complex, we added diamide to a mixture containing hTrx1 and one of the TrxR1 mutants mentioned above. Diamide did not induce the binding of hTrx1 to double Cys497Ser/Sec498Ser mutant of TrxR1. Under identical conditions, hTrx1 could form the complex with other mutants of TrxR1, as shown in Table 4; Figure 4C. These results confirm that Cys189, Cys296 and Cys458 in the non-reduced TrxR1 are not involved in the connection to hTrx1, and that an intermolecular disulfide/selenenylsulfide forms between Cys497/Sec498 of non-reduced TrxR1 and hTrx1 in the presence of diamide. Subsequently, we used the hTrx1 mutant in which Cys73 was substituted by Ala. As shown in Figure 5A, the Cys73Ala mutant of hTrx1 could form a homodimer after incubation with diamide but was no longer able to form the complex with the non-reduced form of TrxR1. Thus, hTrx1-Cys73 is the residue involved in the formation of the TrxR1-hTrx1 complex.

**TABLE 3:** Effect of thiol-reactive reagents on diamide-induced formation of TrxR1-hTrx1 comple**x**.

Thiol-reactive molecule	Formation of diamide-induced TrxR1-hTrx1 complex	
	Before dialysis	After dialysis
DTNB	ND^a^	ND
Aldrithiol^TM^-4	ND	ND
GSSG	Yes	N/T^b^
H_2_O_2_	Yes	N/T
IAM	Yes	N/T
IAA	Yes	N/T

Native TrxR1 was pre-treated with above molecules, respectively. The diamide-induced formation of TrxR1-hTrx1 complex was analyzed before or after removing these molecules *via* dialysis.

^a^ Non-detectable.

^b^ Not Tested.

**TABLE 4:** Identification of residues implicated in covalent connection between the non-reduced TrxR1 and hTrx1 by site-directed mutagenesis**.**

Proteins	Formation of TrxR1-hTrx1 complex
Non-reduced TrxR1 and hTrx1	YES
Double Cys^189^Ser/Sec^498^Cys mutant of TrxR1 and hTrx1	YES
Double Cys^296^Ser/Sec^498^Cys mutant of TrxR1 and hTrx1	YES
Double Cys^458^Ser/Sec^498^Cys mutant of TrxR1 and hTrx1	YES
Tetra Cys^189/296/458^Ser/Sec^498^Cys mutant of TrxR1 and hTrx1	YES
Single Sec^498^Cys mutant of TrxR1 and hTrx1	YES
Single Sec^498^Ser mutant of TrxR1 and hTrx1	YES
DesSec^498^Gly^499^ TrxR1 and hTrx1	YES
Double Cys^497^Ser/Sec^498^Ser mutant of TrxR1 and hTrx1	NO

### 3.5 Stable high-molecular-weight complexes of TrxR1 in human serum

The redox environment of human serum is more oxidizing than the cytosol. Because TrxR1 and hTrx1 are present in human serum and they appear in high-molecular-weight complexes (Wu et al., 2010), we investigated whether the TrxR1 complexes in sera are stable. After approximately 3 months of storage at −70°C, human sera were analyzed for changes in the molecular weight of serum TrxR1 in the presence or absence of DTT. As shown in the left panel of Figure 5B, serum TrxR1 appeared mainly in high-molecular-weight complexes (between 130-170 kDa) when analyzed by non-reducing SDS-PAGE followed by Western blotting. After these samples were treated with DTT, TrxR1 migrated as a monomer (55 kDa) in SDS-PAGE (the right panel of Figure 5B). Because TrxR1 is composed of two identical subunits, each with a molecular weight of approximately 55 kDa, resulting in a molecular weight of approximately 110 kDa for the entire molecule, the high-molecular-weight-complexes large than 110 kDa indicates that TrxR1 also binds to other free thiol-containing molecules through intermolecular disulfide or selenenylsulfide bond(s) under oxidative environment.

## 4 Discussion

### 4.1 Selenenylsulfide-based selective responses of the non-reduced TrxR1 to small-molecule thiol-reactive reagents

Under reducing condition or inside the cell, Sec498-selenol/Cys497-thiol in the C-terminal center of TrxR1 are major targets for anticancer reagents, such as ethaselen (Wang et al., 2012), cisplatin (Anestål and Arnér, 2003), S-250 (Karunanithi et al., 2021), curcumin (Fang et al., 2005), auranofin (Abdalbari and Telleria, 2021) and arsenic trioxide (Lu et al., 2007). These reagents prevent the re-formation of the C-terminal selenenylsulfide between Sec498 and Cys497. It is known that TrxR1 transfers electrons from NADPH to substrates through the following pathway: NADPH → TrxR1 (FAD → active-site disulfide → active-site selenenylsulfide within TrxR1) → Trx1 (or other substrates) (Zhong et al., 2000; Fritz-Wolf et al., 2011). The reversible formation of selenenylsulfide is critical for the catalytic activity of TrxR1 (Lee et al., 2000; Zhong and Holmgren, 2000). In contrast, relatively little is known about reaction properties of the non-reduced TrxR1 under oxidative conditions or outside the cells.

Inspired by the way the reaction of the non-reduced TrxR1 with DTNB generated TNB^−^ in the absence of NADPH (Figure 2B), we report use of small thiol-reactive molecules as models for a series of non-reduced TrxR1 reactions under oxidative conditions. The operating principle of this chemistry is that selenenylsulfide is reactive towards electrophilic molecules, thus accelerating the exchange between the different species.

The current study shows that the non-reduced form of TrxR1 displays variations in reactivity with the different thiol-reactive reagents. The differences are a ∼50% decrease in TrxR1 activity with GSNO treatment, a ∼73% decrease in TrxR1 activity with DTNB treatment, whereas no significant decrease in TrxR1 activity after treated with buffer, H_2_O_2_, diamide, GSSG, Px-12, or IAM (Figure 1A). Of particular note, although Px-12 has no effect on the activity of the non-reduced TrxR1, it can inhibit the activity of NADPH-reduced TrxR1 (Figure 1B), which is similar to IAM (Zhong et al., 1998), indicating that their reaction targets are free thiol or selenol groups. Following this reasoning, we predict that the selenenylsulfide critically influences the inhibitory or non-inhibitory nature of these small-molecule thiol-reactive reagents.

### 4.2 Cleavage of dynamic S-S bond in DTNB by the selenenylsulfide in the non-reduced TrxR1

Using DTNB as a model and point mutation technology, we confirm that only TrxR1 mutant without C-terminal Cys497 and Sec498 residues did not result in the release of TNB^−^ from DTNB (Figure 2B). Apparently, the presence of the selenenylsulfide in the non-reduced TrxR1 produces a positive effect on the release of TNB^−^ from DTNB. We then checked whether the purified TrxR1 might coexist with a Sec498Cys mutant of TrxR1, since under selenium-deficient conditions, the Sec498Cys mutant of TrxR1 was found to be synthesized (Lu et al., 2009). This mutant maintains the C-terminal Cys couple in a dithiol form (Sandalova et al., 2001). The thiol can either react with DTNB or be alkylated by IAM. Our purified TrxR1 was incubated with IAM, followed by dialysis to remove excess IAM. Unlike DTNB-pretreated non-reduced TrxR1, IAM-pretreated non-reduced form of TrxR1 was highly active (Figure 1A; Table 1). These results exclude the presence of Sec498Cys mutant in our purified TrxR1, and support that the ability of the non-reduced TrxR1 to react with DTNB is provided by the selenenylsulfide bond. In the absence of NADPH, DTNB seems to be an alkylating reagent rather than a substrate of TrxR1.

### 4.3 Theoretical mechanism for the selenenylsulfide to attack the S-S bond of DTNB or the S-N bond of GSNO

Our observations can be rationalized by understanding the type of bonds that participate in reactions with selenenylsulfide bond (S-Se bond), which becomes highly polarized (R2^−^ in Figure 3) under our experimental conditions at pH 7.5; the electron density moves away from the S atom towards the Se atom. This step results in a nucleophilic Se atom (electron-rich Se). However, DTNB contains a highly electrophilic disulfide because of the presence of symmetrical 2-nitrobenzoate groups bonded to each S atom with a good leaving group of TNB^−^ (Winther and Thorpe, 2014). GSNO has a polar S-N bond with electrophilic properties. Accordingly, in the presence of DTNB, electron transfer from R2^−^ in Figure 3A to DTNB is easy; that is, the Se serves as the electron donor for reducing DTNB and forming a mixed Se-S bond and released TNB^−^ (Figure 3C). Similarly, the Se acts as a nucleophile, attacking the nitroso group of GSNO and forming a Se-N bond (Figure 3C) through a mechanism similar to transnitrosation (Shimada et al., 2004; Barglow et al., 2011; Broniowska et al., 2013). This mechanism can probably be extended to hTrx1-dependent inhibition of GSNO or DTNB on TrxR1 in the presence of NADPH (Luthman and Holmgren, 1982; Nikitovic and Holmgren, 1996), which involves thioalkylation of a critical cysteine residue (Kirkpatrick et al., 1998). Since the selenenylsulfide is re-formed after the reduction of hTrx1 by selenolthiol (Zhong et al., 2000), the re-formed selenenylsulfide might react with DTNB or GSNO, at least partially depriving TrxR1 activity. By contrast, GSSG contains a symmetrical, non-polarized disulfide, and H_2_O_2_ is also a non-polar molecule. Both molecules have little ability to promote electron release from the S-Se bond (R2^−^ in Figure 3A). Thus, it is difficult to break the selenenylsulfide using GSSG or H_2_O_2_. This chemical property may explain why pretreatment with GSSG or H_2_O_2_ causes no effect on either the activity of the non-reduced TrxR1 or the ability of the non-reduced TrxR1 to form TrxR1-hTrx1 complexes in the presence of diamide (Figure 1A, lane 6 in Figure 4B; Table 3). Thus, the general principle of the selenenylsulfide sensing in the non-reduced TrxR1 is the process of opening selenenylsulfide bond in response to the oxidizing reagents with a highly electrophilic active species.

### 4.4 Proposed mechanism for electrophile diamide to induce the mixed disulfide between the non-reduced TrxR1 and hTrx1

Following the above reasoning, we speculated that the TrxR1 would also be unstable in the presence of the electrophile diamide. To test this speculation, diamide was mixed with the non-reduced form of TrxR1. Under these conditions, TrxR1 displayed a ∼86.4% decrease in its ability to transfer electrons from NADPH to hTrx1 (Table 1). This decrease was no longer observed when diamide was removed *via* dialysis (Figure 1A; Table 1). This finding may be due to that diamide inhibits hTrx1 *via* inducing a second disulfide, dimer as well as multimers (Hashemy and Holmgren, 2008), which is not a substrate of TrxR1. It is worth noting that diamide did not cause covalent bonding between two TrxR1 subunits (lane 4 in Figure 4A and lane 5 in Figure 4B). Concerning these results, the non-reduced TrxR1 with selenenylsulfide seems to be a “diamide-protective” form.

However, incubation of the non-reduced TrxR1 with hTrx1 in the presence of diamide caused the formation of the TrxR1-hTrx1 complex (lanes 2,3,5 in Figure 4A, lane 3 in Figure 4B). The potential mechanism underlying diamide-induced formation of the TrxR1-hTrx1 complex is somewhat different from that involved when selenenylsulfide reacts with DTNB or GSNO.

**FIGURE 6:**
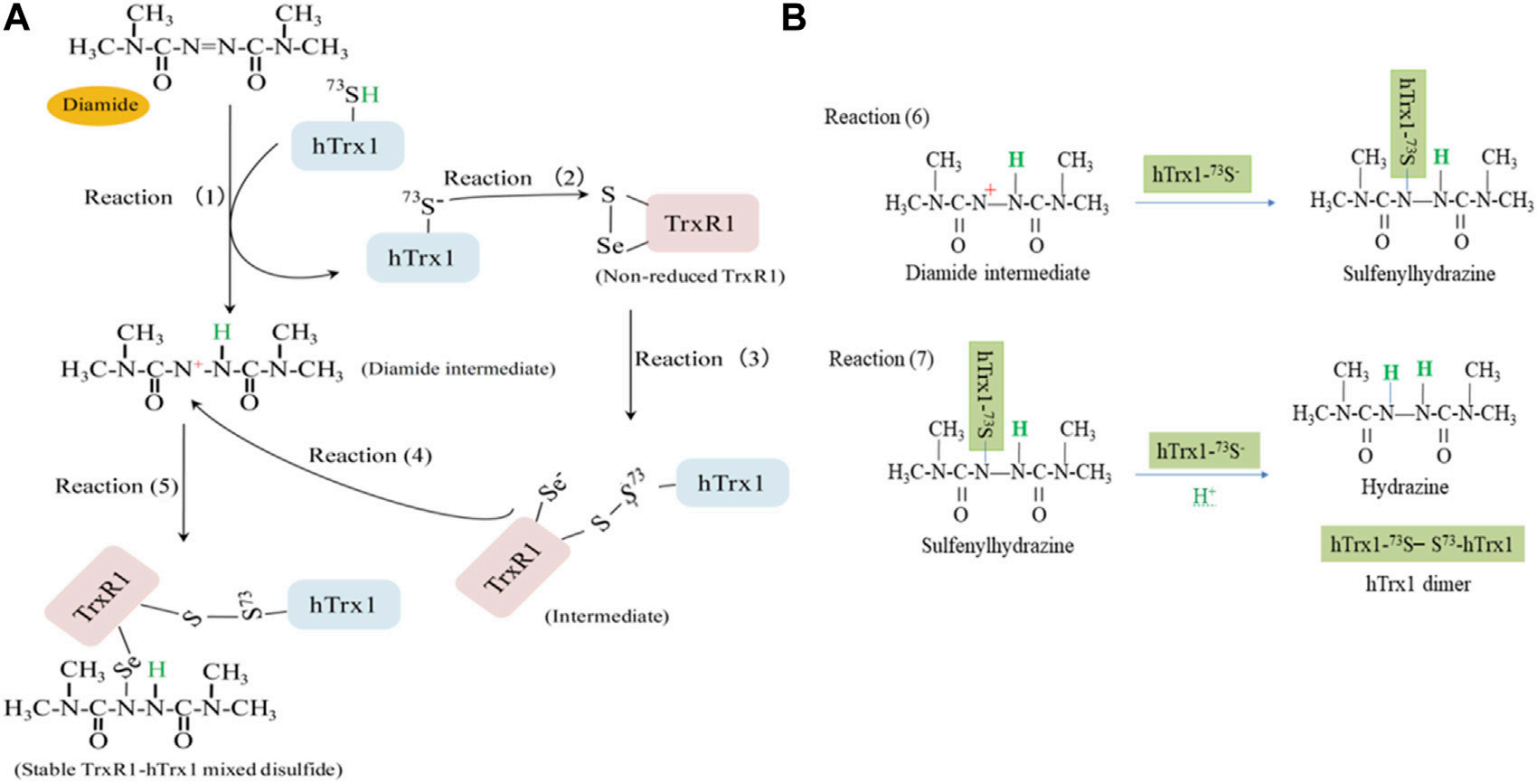
A proposed mechanism for diamide to induce a stable TrxR1-hTrx1 complex. **(A)** At pH 7.5, selenenylsulfide has the S atom with a partial positive charge. Electrophile diamide causes hTrx1-Cys73 thiolation (reaction 1). The thiolate anion attacks the S atom of selenenylsulfide (reaction 2), resulting in the formation of a mixed disulfide between TrxR1-Cys497 and hTrx1-Cys73 (reaction 3). After receiving one proton from hTrx1-Cys73, the -N=N- bond of diamide becomes a polarized N-N bond. The partial positive charge on the N atom can covalently attach to selenolate (reaction 4), forming a stable TrxR1-hTrx1 complex (reaction 5). **(B)** Reaction of diamide intermediate from reaction (1) with hTrx1-Cys73 to form hTrx1 dimer (reactions 6 and 7).

After summarizing our experimental results, we have postulated the following mechanism. Electrophilic diamide may promote proton release from the thiol of hTrx1-Cys73 to form a thiolate anion (reaction 1 in Figure 6A). The released proton is added to the -N=N- bond of diamide, forming a reactive intermediate with a polarized N-N bond (diamide intermediate in Figure 6A). The resulting thiolate of hTrx1-Cys73 would attack the S atom of the selenenylsulfide (reaction 2 in Figure 6A) to form an intermediate complex of TrxR1-hTrx1 (reaction 3 in Figure 6A). This reaction can happen easily because of the superior leaving group ability of a selenol compared to thiol (Canal-Martín and Pérez-Fernández, 2021). Consequently, the resulting diamide intermediate may react with the selenolate anion in the intermediate complex of TrxR1-hTrx1 (reaction 4 in Figure 6A) to form a stable TrxR1-hTrx1 complex (reaction 5 in Figure 6A). This is consistent with the observation that the Cys73Ala mutant of hTrx1 no longer combines with TrxR1 (lane 5 in Figure 5A). In addition, the diamide intermediate may react with the thiolate anion of hTrx1-Cys73 to form sulfenylhydrazine (reaction 6 in Figure 6B). Then, sulfenylhydrazine may react with thiolate anion of another hTrx1-Cys73, generating one hTrx1 homodimer and a hydrazine (reaction 7 in Figure 6B). The presence of TrxR1-hTrx1 complex and hTrx1 homodimer in the reaction mixture was confirmed by non-reducing SDS-PAGE (Figures 4A, B), on which the molecular weight of sulfenylhydrazine was expected to be similar to that of hTrx1 monomer. Diamide is an important functional group present in certain drugs (Ding et al., 2012; Abdelrahman et al., 2019). The effective effect of diamide on the non-reduced TrxR1 requires an additional molecule with free thiol group. The current findings may deepen our insight into these drugs’ mechanism of actions.

As shown in Figure 5A, Cys73 is the key site for hTrx1 to form the TrxR1-hTrx1 complex, whereas Cys73Ala mutant still appeared as a homodimer. Similarly, Cys73Ser mutant could also form a homodimer (Lou et al., 2017). It seems to be correlated with structural Cys62/69. hTrx1 contains two active site Cys32 and Cys35 residues as well as three structural Cys62, Cys69 and Cys73 residues (Hashemy and Holmgren, 2008). Under different stress conditions, disulfide bond forms in the active site (Cys32 and Cys35), Cys62 and Cys73 are modified by S-nitrosylation (Hashemy and Holmgren, 2008) or S-palmitoylation (Qin et al., 2015). All of there results indicates that Cys62 might be involved in the formation of the homodimer between two Cys73Ala mutant monomers.

### 4.5 TrxR1 from human serum samples exists in high-molecular-weight forms with potential activity

To gain more information about the status of extracellular TrxR1 in a biological system, we obtained human serum samples, which were the remaining parts of the previous experiments. TrxR1 is present in human plasma or serum (Söderberg et al., 2000; Wu et al., 2010), where there is a lower NADPH/NADP^+^ ratio as compared to that in the cytosol. The environment of serum allows the non-reduced form of TrxR1 to dominate. In human serum, TrxR1 existed in high-molecular-weight species without DTT and appeared to be monomer size (55 kDa) with DTT (Figure 5B) (Wu et al., 2010), most likely owing to the following mechanism. Serum TrxR1 exists in a non-reduced form that contains selenenylsulfide. The latter participates in connecting to other molecules *via* mixed disulfide/selenenylsulfide in a non-reducing environment. This indicates that serum TrxR1 complexes with high-molecular-weight are stable, reducible and potentially active in transferring electrons from NADPH to the substrate (Wu et al., 2010). A consequence of the existed TrxR1 complex in serum seems to be protection of the non-reduced TrxR1 from responding to certain electrophilic reagents. However, release of appropriate reducing agents by cells can break the reducible linkages and restore the reactivity of the non-reduced TrxR1.

In short, the actions of electrophilic reagents on the non-reduced TrxR1 are correlated with their structure. The inhibitory action of DTNB or GSNO is mediated, at least in part, in this manner by breaking the selenenylsulfide to form mixed Se-S bond or Se-N bond (Figure 3C) under oxidative conditions, which can lead to inactivation of TrxR1 (Figure 1A; Table 1). Those oxidants, such as GSSG and H_2_O_2_, lack the ability to open the selenenylsulfide of the non-reduced TrxR1. Similar to results with DTNB and GSNO, the nucleophilic attack of the Se-S bond on the Au(I) center in auranofin was most recently reported (Dos Santos and Paschoal, 2024). Their explanation coincides with the mechanism we proposed, that is, the nucleophilic attack of the Se-S bond on the a highly electrophilic species. From another point of view, the inhibition of TrxR1 by GSNO or DTNB in the presence of hTrx1 (Luthman and Holmgren, 1982; Nikitovic and Holmgren, 1996) also involves hTrx1-Cys73 modification by GSNO (Barglow et al., 2011) or DTNB (Zhong’s observation). Oxidant diamide, an electrophile, may modify non-reduced TrxR1 but requires reductive activation, for example, with thiol (Figure 6).

Various disorders, including primary liver cancer (Wu et al., 2021) and breast cancer (Bhatia et al., 2016), are found to be significant elevation of TrxR1 to cope with oxidative stress. Since ROS level in cancer cells is higher than that in normal cells, the non-reduced form of TrxR1 can also increase in cancer cells. Thus, amelioration of non-reduced TrxR1’s reactivity has direct therapeutic implications. Since the non-reduced TrxR1 is susceptible to the compounds containing dynamic covalent bond or a highly electrophilic species, the intrinsic reactivity of these active species is an important consideration in the design of small-molecule inhibitors for the non-reduced form of TrxR1.

## Data Availability

The datasets presented in this study can be found in online repositories. The names of the repository/repositories and accession number(s) can be found in the article/Supplementary Material.

## Supplementary material

SUPPLEMENTARY FIGURE S1 Molecular model of non-reduced TrxR1. The crystallographic data were from the Molecular Modeling Database (MMDB ID: 68283) or Protein Data Bank (PDB ID: 3EAO). **(A, C)** The spheres representation. **(B, D)** The surface representation. The Cys residues and Sec residue present in the X-ray structure are shown as yellow spheres.

## Abbreviations

TrxR1, cytosolic thioredoxin reductase; hTrx1, human cytosolic thioredoxin; MMP, matrix metalloproteinases; TIMP, inhibiting tissue inhibitors of MMPs; DTNB, 5,5′-Dithiobis-(2-nitrobenzoic acid); Diamide, Diazinedicarboxylic acid bis(N,N-dimethylamide); GSH, reduced glutathione; GSSG, oxidized glutathione; H2O2, hydrogen peroxide; IAM, iodoacetamide; IAA, iodoacetic acid.
