# Supplementary figures and images for: The C-terminal selenenylsulfide of extracellular/non-reduced thioredoxin reductase endows this protein with selectivity to small-molecule electrophilic reagents under oxidative conditions
Source: Front Mol Biosci. 2024 Mar 8;11:1274850. doi: 10.3389/fmolb.2024.1274850 (PMC10957665; doi:10.3389/fmolb.2024.1274850)

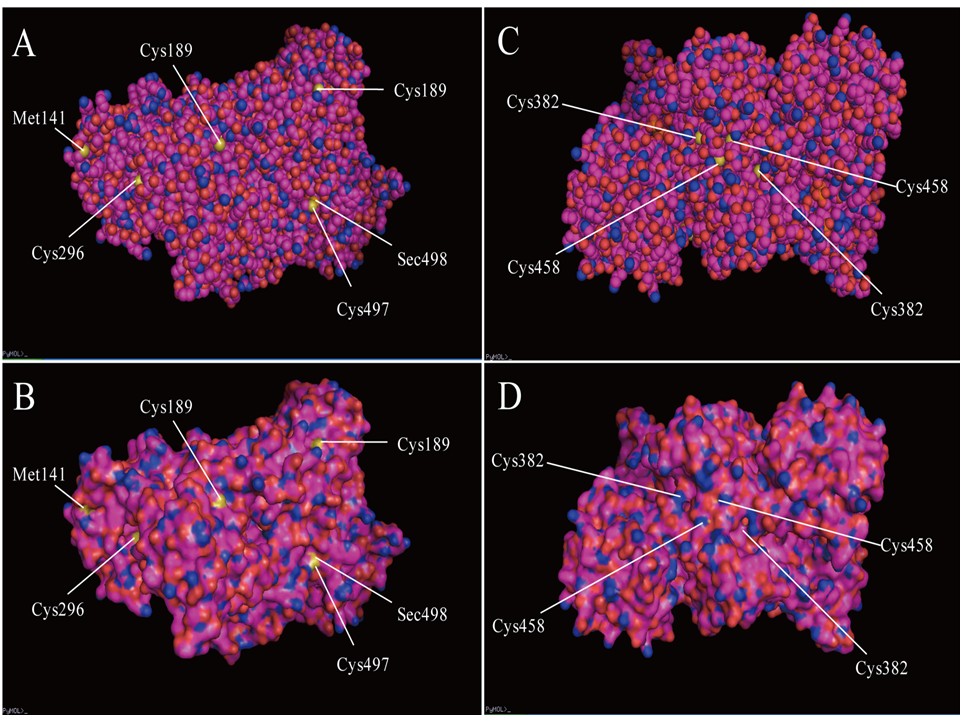

Supplement: Supplementary file 1 [file Image1.TIF]
