# Supplementary material for: The C-terminal selenenylsulfide of extracellular/non-reduced thioredoxin reductase endows this protein with selectivity to small-molecule electrophilic reagents under oxidative conditions
Source: Front Mol Biosci. 2024 Mar 8;11:1274850. doi: 10.3389/fmolb.2024.1274850 (PMC10957665; doi:10.3389/fmolb.2024.1274850)
Supplement: Supplementary file 2 [file Table1.DOC]

**Supplementary Table 1:** Structure of thiol-reactive molecules

| **Name** | **Structure** |
| --- | --- |
| H_2_O_2_ | 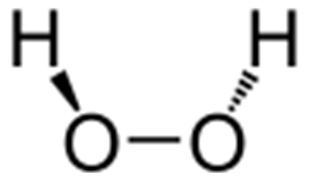 |
| GSSG | 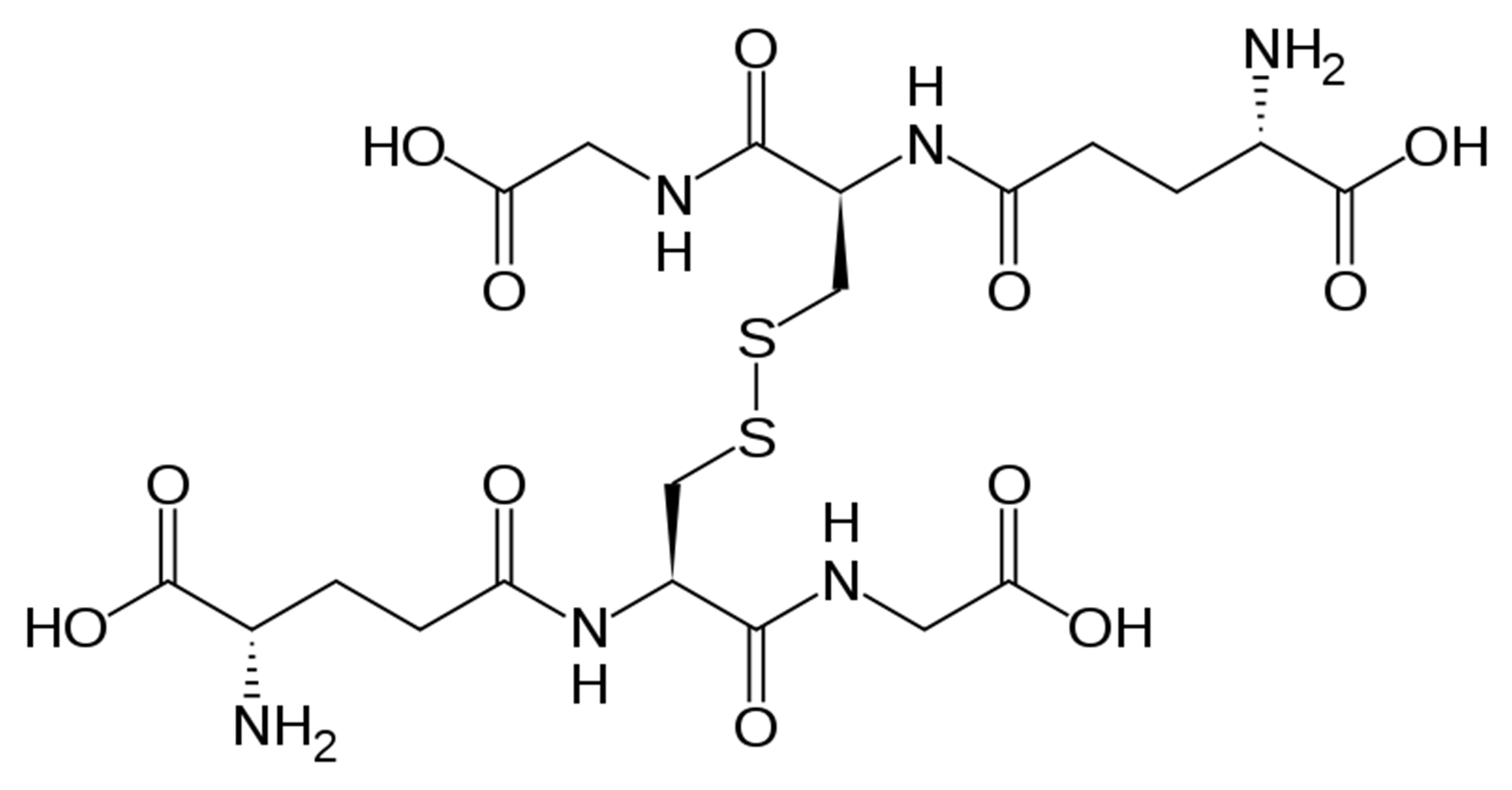 |
| IAM/IAA | 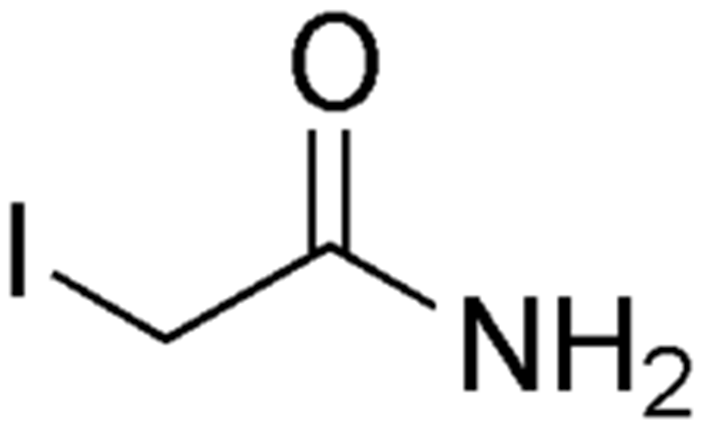 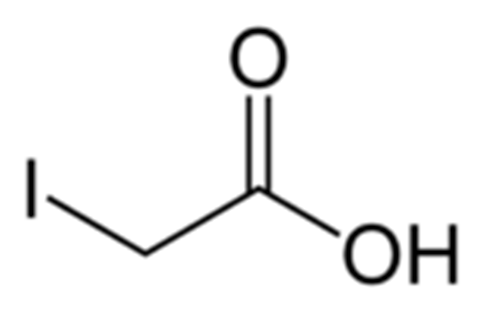 |
| PX-12 | 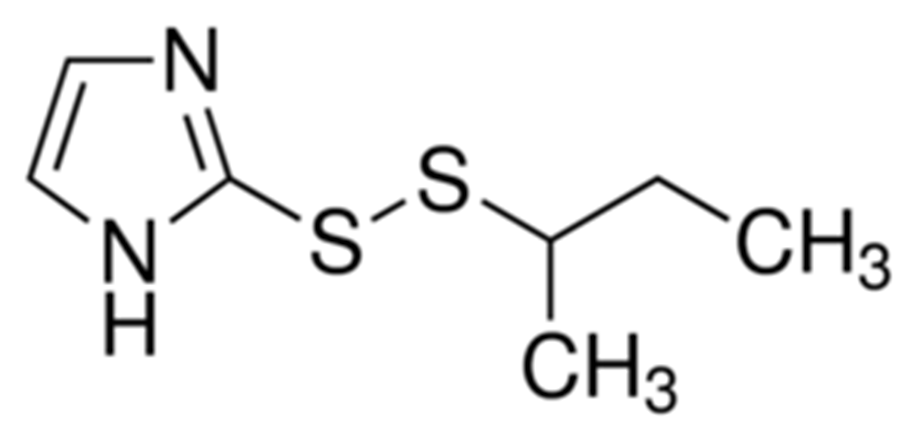 |
| Diamide | 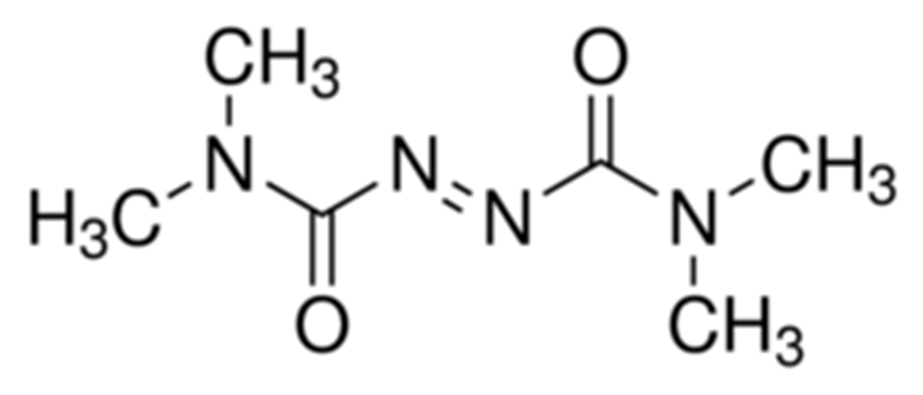 |
| GSNO | 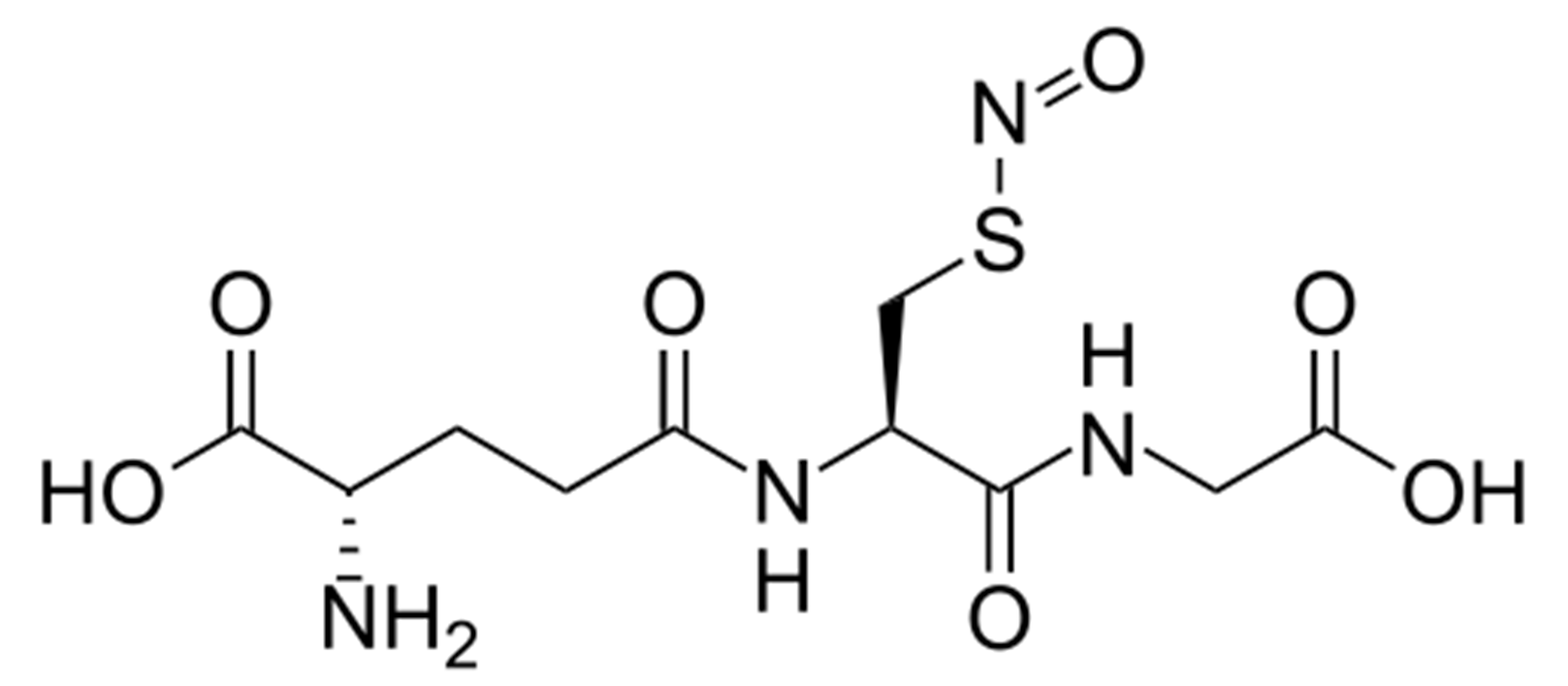 |
| DTNB | 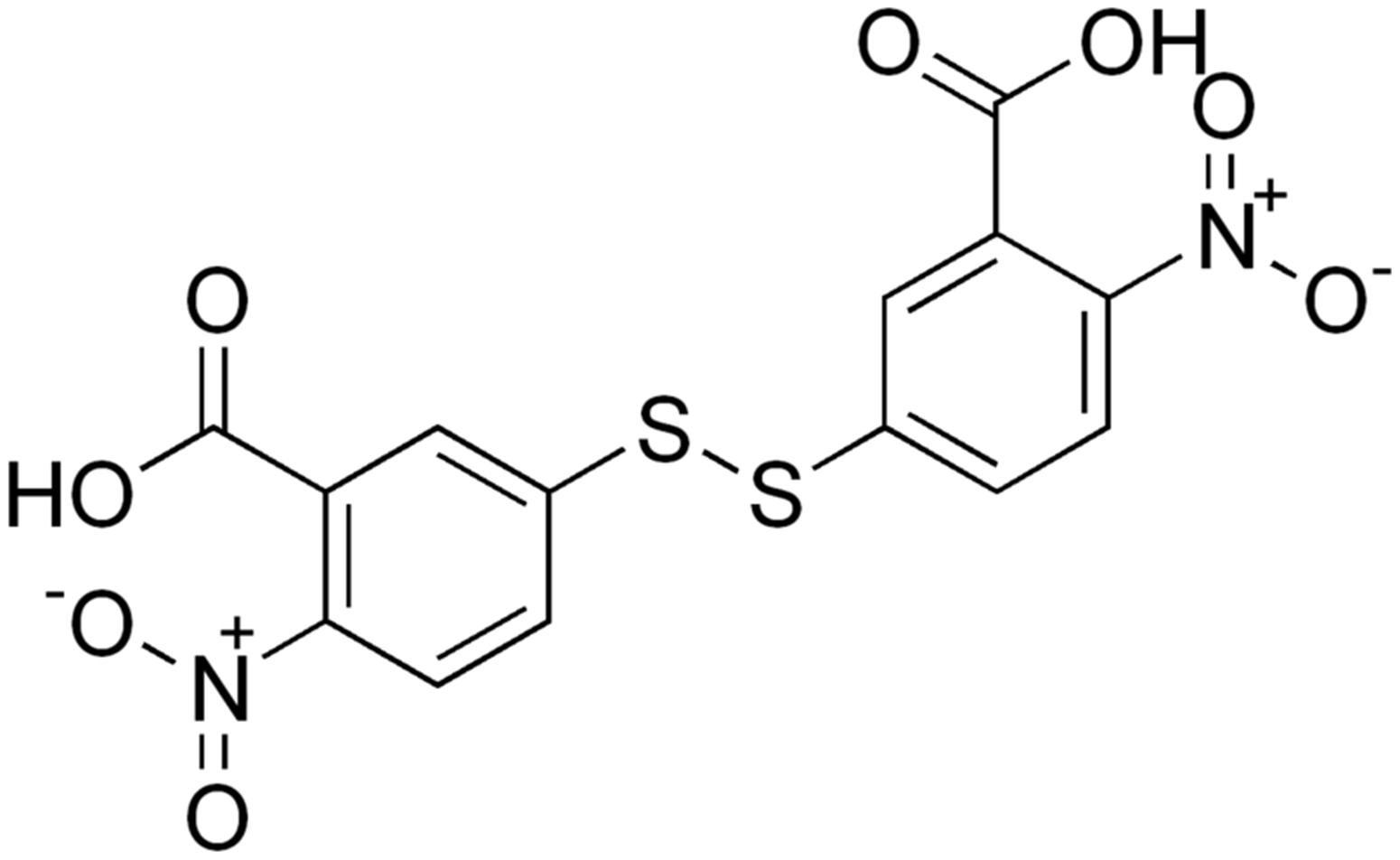 |
| Aldrithiol^TM^-4 | 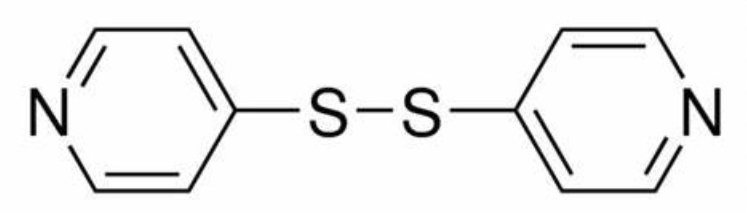 |
